# Capsaicin suppresses ciliary function, while inducing permeability in bronchial epithelial cell cultures of COPD patients

**DOI:** 10.3389/fphar.2022.996046

**Published:** 2022-10-06

**Authors:** Halil Ibrahim Toy, Abdullah Burak Yildiz, Demet Tasdemir Kahraman, Sedat Ilhan, Oner Dikensoy, Hasan Bayram

**Affiliations:** ^1^ Gaziantep University School of Medicine, Respiratory Research Laboratory, Gaziantep, Türkiye; ^2^ Izmir Biomedicine and Genome Center, Izmir, Türkiye; ^3^ Izmir International Biomedicine and Genome Institute, Dokuz Eylül University, Izmir, Türkiye; ^4^ Department of Epidemiology and Cancer Control, St. Jude Childrens Research Hospital, Memphis, TN, United States; ^5^ Koc University School of Medicine, Istanbul, Türkiye; ^6^ Gaziantep University, Faculty of Medicine, Department of Medical Biochemistry, Gaziantep, Türkiye; ^7^ Gaziantep University, Institute of Health Sciences, Department of Respiratory Biology, Gaziantep, Türkiye; ^8^ Department of Chest Diseases, Gaziantep University School of Medicine, Gaziantep, Türkiye; ^9^ Department of Pulmonary Medicine, Koc University School of Medicine, Istanbul, Türkiye; ^10^ Koc University Research Centre for Translational Medicine (KUTTAM), Koç University, Istanbul, Türkiye

**Keywords:** tear gas, ciliary beat frequency (CBF), transepithelial electrical resistance (TEER), formoterol, roflumilast, airways, cytokine

## Abstract

**Take Home Message:** Capsaicin modified inflammatory response and caused toxicity in bronchial epithelial cultures from patients with COPD. More importantly, capsaicin decreased ciliary beat frequency and induced epithelial permeability and these effects were partially prevented by formoterol and roflumilast.

Tear gas is widely used to halt mass demonstrations. Studies have reported its adverse effects on multiple organ systems; however, its effect on individuals with chronic respiratory diseases and the underlying mechanisms of these effects are unclear. For the first time in the literature, we investigated the effects of capsaicin, the active ingredient of tear gas, on bronchial epithelial cell (BEC) cultures obtained from well-characterized groups of nonsmokers, smokers, and patients with chronic obstructive pulmonary disease (COPD). BEC cultures were incubated with 50-500 μM capsaicin in the absence and presence of formoterol (1μM) and roflumilast (0.1 μM) for 24 h. Ciliary beat frequency (CBF) and transepithelial electrical resistance (TEER) were assessed at T1/4, T1/2, T1, T2, T4, T6, and T24 h, whereas the release of granulocyte-macrophage colony-stimulating factor (GM-CSF), interleukin (IL)-8, and lactate dehydrogenase (LDH) was measured at T24 h. Capsaicin (250 µM) significantly decreased CBF of all BEC cultures from T1/4 h to T24 h (p<0.05). Formoterol significantly prevented decreases in CBF induced by capsaicin. Higher concentrations of capsaicin (250-500 μM) significantly reduced TEER of BECs from nonsmokers (T2-T24 h), smokers (T24 h) and COPD patients (T2 and T24 h), which was partially prevented by roflumilast. Capsaicin (500 μM) decreased release of IL-8 (p<0.0001) and GM-CSF (p<0.05) while inducing release of LDH in BECs (p<0.05), and this was more prominent in BEC from patients with COPD. In conclusion, our findings demonstrate that capsaicin can suppress ciliary activity and cytokine release from BECs, induce BEC culture permeability and cellular toxicity and that these effects can be partially prevented by formoterol and roflumilast.

## Introduction

Tear gas, one of the most common riot agents, has been being widely used to halt mass demonstrations (Toprak et al., 2015; Yeung and Tang, 2015). Studies have suggested that exposure to tear gas may cause toxic effects in respiratory and other systems (Yeung and Tang, 2015; Rothenberg et al., 2016). Airway mucosa is highly sensitive to capsaicin, the main active ingredient of tear gas, and its exposure causes intense irritation (Stjärne et al., 1991). Studies have reported that capsaicin leads to burning sensation, cough, difficulty in speaking, choking, chest tightness, haemoptysis, mucus secretion, and inflammation in the airways (Yeung and Tang, 2015; Rothenberg et al., 2016). Capsaicin was also reported to cause increased airway resistance (Johansson et al., 2019), pulmonary oedema, apnoea, and respiratory arrest (Tuorinsky et al., 2008).

Studies have suggested that subjects with existing respiratory diseases including asthma, reactive airway disease and chronic bronchitis are more susceptible to tear gas exposure (Rothenberg et al., 2016). Capsaicin reduced forced expiratory volume in the first second (FEV_1_) (Johansson et al., 2019), even led to death in some asthmatics (Steffee et al., 1995). Tear gas exposure led to a greater decline in both FEV_1_ and forced vital capacity (FVC) in smokers, as compared to non-smokers (Ilgaz et al., 2019).

Oleoresin capsicum (OC), is a concentrated oily extract from the seeds of the Solanaceae family chili pepper plants *C. annuum*, *C. frutescens* and *C. chinense* (Yeung and Tang, 2015). Human studies have reported that nasal sprays of capsaicin cause increases in the leukocyte counts and albumin and lysozyme levels (Sanico et al., 1997). Furthermore, capsaicin induced a cough reflex and increased nerve growth factor (NGF) in nasal lavage fluids of patients with asthma-like symptoms (Millqvist, 2000). In controlled mild atopic asthmatic patients, prior bronchoconstriction showed an increased capsaicin-evoked cough by increasing the activation of capsaicin-responsive airway nerves (Satia et al., 2017).

Animal studies have demonstrated that OC decreased the ciliary beat frequency (CBF) of mouse tracheal epithelial cells, which was prevented by methylene blue (Delamanche et al., 2001). Mice developed acute inflammation in the lungs and airway cell injury depending on the dose of capsanoids (Reilly et al., 2003). Other studies have reported increases in the levels of plasma total IgE, eosinophils, and Th2 cytokines, including interleukin (IL)-4, IL-5, and IL-13, together with a prolonged expiration duration with a rapid shallow breathing pattern, hypotension, bradycardia, increased airway resistance and decreased lung compliance in rats (Han et al., 2017; Lee and Kuo, 2017; Chen et al., 2020). A limited number of *in vitro* studies have reported that capsanoids cause cellular death and IL-6 release from human lung epithelial cell lines (BEAS-2B and A549) and liver cell lines (HepG2) and that lung cells are more sensitive to these deleterious effects than liver cells (Reilly et al., 2003).

In the present study, we investigated the effects of capsaicin on primary human bronchial epithelial cells (BEC) cultured from non-smokers, smokers, and smoker patients with chronic obstructive pulmonary disease (COPD), and found that capsaicin can show detrimental effects on these cells.

## Materials and methods

### Study patients

Twenty-five patients (21 male and 4 female) with a mean age of 64 years (18–80 years) participated in the study. Of these, 5, 13 and 7 patients were non-smokers, smokers, and smokers with COPD (COPD patients), respectively, according to the guidelines from the Global Initiative for Obstructive Lung Disease (GOLD) (Global Initiative for Chronic Obstructive Lung Disease, 2022). The demographics of the study subjects are presented in Table 1. None of the patients had upper or lower respiratory tract infections within 1 month prior to the study. The study was approved by The Ethics Committee of Gaziantep University (2014/346), informed written consent was obtained from the study patients, and experiments adhered to the principles set out in the Declaration of Helsinki.

**TABLE 1:** | Demographics of patients.

	Non-smokers	Smokers	COPD
Sex	4/1 (F/M)	13 (M)	7 (M)
Age (mean, range)	53 (18–80)	66 (51–80)	66 (57–75)
Smoking History (packs/year)	-	55.9 ± 7.8	67.1 ± 16
FEV1 (%, mean ± SEM)	96.2 ± 14.7	94.2 ± 4.2	61.9 ± 4.5 *++
FVC (%, mean ± SEM)	98.2 ± 14.4	99.7 ± 3.7	84.9 ± 8
FEV1/FVC (mean ± SEM)	80.2 ± 1.7	76.5 ± 2	56.6 ± 5.1 **+

(*p < 0.05 and **p < 0.01 vs. non-smokers; +*p* < 0.01 and ++*p* < 0.001 vs. smokers). FEV_1_: Forced Expiratory Volume in the first second, FVC: Forced Vital Capacity, SEM: Standard Error of Mean, COPD: Chronic obstructive pulmonary disease, F: Female, M: Male.

### Bronchial tissue

Bronchial tissue was obtained from patients who had a lobectomy or pneumonectomy for various reasons, at the Thoracic Surgery Clinic of Gaziantep University, Şahinbey Research and Training Hospital. The bronchial explant that appeared free of tumour and was considered normal by the pathologist was placed into ice-cold Medium 199 (Sigma, Interlab, Türkiye) and transferred to the laboratory for tissue culture processing.

### Isolation, culture, and identification of bronchial epithelial cells

BEC cultures were obtained using an explant cell culture technique developed by Devalia et al. and described in detail elsewhere (Devalia et al., 1990; Gogebakan et al., 2014). Cultures were established on 6-cm diameter Falcon® Primaria^TM^ plastic culture dishes (Becton Dickinson, Türkiye) or into 9-mm-diameter Falcon® Cell Culture Inserts with 0.45-mm pore size microporous membranes (Becton Dickinson) as air liquid interface (ALI) cultures for CBF and TEER studies, respectively (Bayram et al., 1998; Bayram et al., 2002).

### Preparation of capsaicin, formoterol and roflumilast solutions

Capsaicin was dissolved in ethanol (Sigma) and prepared in serum-free medium containing medium-199 and antibiotics/antimycotic (SF) at concentrations of 50, 125, 250 and 500 µM for experiments Formoterol and roflumilast (Tocris, Medsantek, Türkiye) were dissolved in dimethyl sulfoxide (DMSO), and concentrations of 1 μM formoterol and 0.1 µM roflumilast were added to solutions containing 250 μM and 500 µM capsaicin for CBF and TEER experiments, respectively. These specific concentrations of capsaicin (Tsukura et al., 2007), formoterol (Erjefält and Persson, 1991; Anderson, 1993), and roflumilast (Milara et al., 2012; Schmid et al., 2015) were chosen based on the existing literature, and our ongoing studies investigating effects of formoterol and roflumilast on CBF in primary BECs.

### Measurement of CBF and TEER in BECs incubated with capsaicin in the absence and presence of formoterol or roflumilast

CBF was measured using a Sisson-Ammons image analysis system (SAVA) (Ammons Engineering, Clio, MI, United States ). CBF was measured at 1/4, 0.5, 1, 2, 4, 6, and 24 h in the absence or presence of 50, 125 and 250 µM capsaicin, 1 µM formoterol or 0.1 µM roflumilast. CBF in each culture was calculated as the mean of 5 areas within the culture at baseline and at each time point during incubation; the effect of treatment was expressed as the percentage change from the baseline.

Confluent BEC cultures established under ALI conditions were incubated with 0, 50, 250, or 500 µM capsaicin both from apical and basolateral surfaces in the absence and presence of 1 µM formoterol or 0.1 µM roflumilast, and TEER was measured at t0, t1, t2, t4, t6 and t24 h using an EVOM2™ microvolt-ohm metre (WPI World Precision Instruments, Germany). TEER was expressed as the percent change from baseline. At the end of the measurements, the media inside inserts and wells were collected and stored at -80°C for the analysis of GM-CSF, IL-8 and LDH. The culture membranes were detached from the insert and stored at -80°C for total protein analysis.

### Analysis of IL-8, GM-CSF, LDH, and total cellular protein

Levels of IL-8, GM-CSF and LDH were analysed using ELISA kits (R&D System Duoset, Starmed, Türkiye) and LDH kits (Sigma) according to the manufacturer’s instructions. Total cellular protein was measured using the Qubit protein analysis kit (Invitrogen, Medsantek, Türkiye). The levels of cytokines and LDH were expressed as pg of cytokine and milliunits (mIU) of LDH/µg of cellular protein.

### Statistical analysis

Data were tested for normality with D’Agostino and Pearson omnibus normality test, and continuous variables were compared using one-way variance analysis, ANOVA/Dunnett’s multiple comparison tests or Kruskal–Wallis/Dunn’s multiple comparison tests. The results are expressed as means ± SEM or medians ± interquartile ranges (Q1 and Q3) and lower and upper ranges. *p* ≤ 0.05 was regarded as significant. Statistical analysis was performed using PRISM version 8 (GraphPad Software Inc., San Diego, CA, United States ).

## Results

### Effects of capsaicin with and without formoterol or roflumilast on the CBF of BECs

**FIGURE 1:**
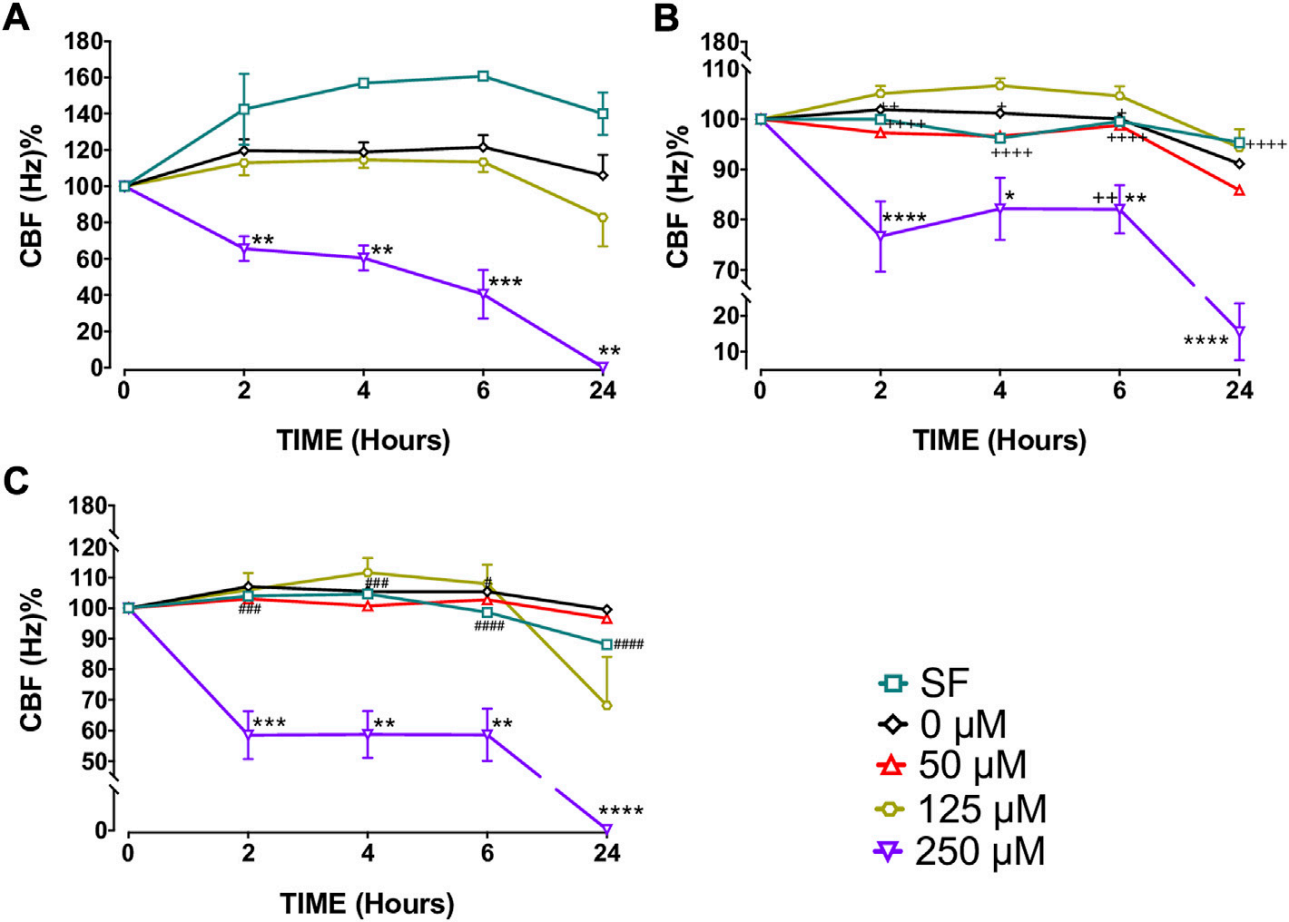
Effects of capsaicin (50, 125 and 250 µM) on ciliary beat frequency (CBF) of bronchial epithelial cell (BEC) cultures of non-smokers **(A)**, smokers **(B)** and patients with COPD **(C)**. Mean ± SEM is displayed for non-smoker (*N* = 5), smoker (*N* = 13) and patients with COPD (*N* = 7). (*p < 0.05, **p < 0.01, ***p < 0.001, ****p < 0.0001 vs. 0 μM capsaicin; +*p* < 0.05, ++*p* < 0.01, ++++*p* < 0.0001 vs. non-smokers; #*p* < 0.05, ###*p* < 0.001, ####*p* < 0.0001 vs. smokers). SF, serum free treated cells; 0 µM indicates ethanol, solvent for capsaicin.

Capsaicin (250 µM), significantly decreased CBF in BEC cultures from the three study groups at T2, T4, T6, and T24 h compared with BEC cultures from the control group, 0 µM (*p* < 0.05–0.0001; Figure 1 and Supplementary Figure S1, Supplementary Table S1). Following T6 incubation with 250 µM capsaicin, the CBF of smokers’ BECs was higher than that of non-smoker cultures. (*p* < 0.01; Figure 1 and Supplementary Figure S1, Supplementary Table S1). Formoterol (1 µM) significantly prevented the decreasing effect of 250 μM capsaicin on the CBF of smokers’ BEC cultures at T¼, T½, T1, and T2 h (*p* < 0.05). However, roflumilast (0.1 µM) showed no effect (Figure 3A, Supplementary Table S1). Under normal baseline conditions CBF of BEC cultures from patients with COPD was significantly higher than both non-smokers and smokers at T0 and T2 (Supplementary Figure S2).

### Effect of capsaicin with and without formoterol or roflumilast on TEER in BECs

**FIGURE 2:**
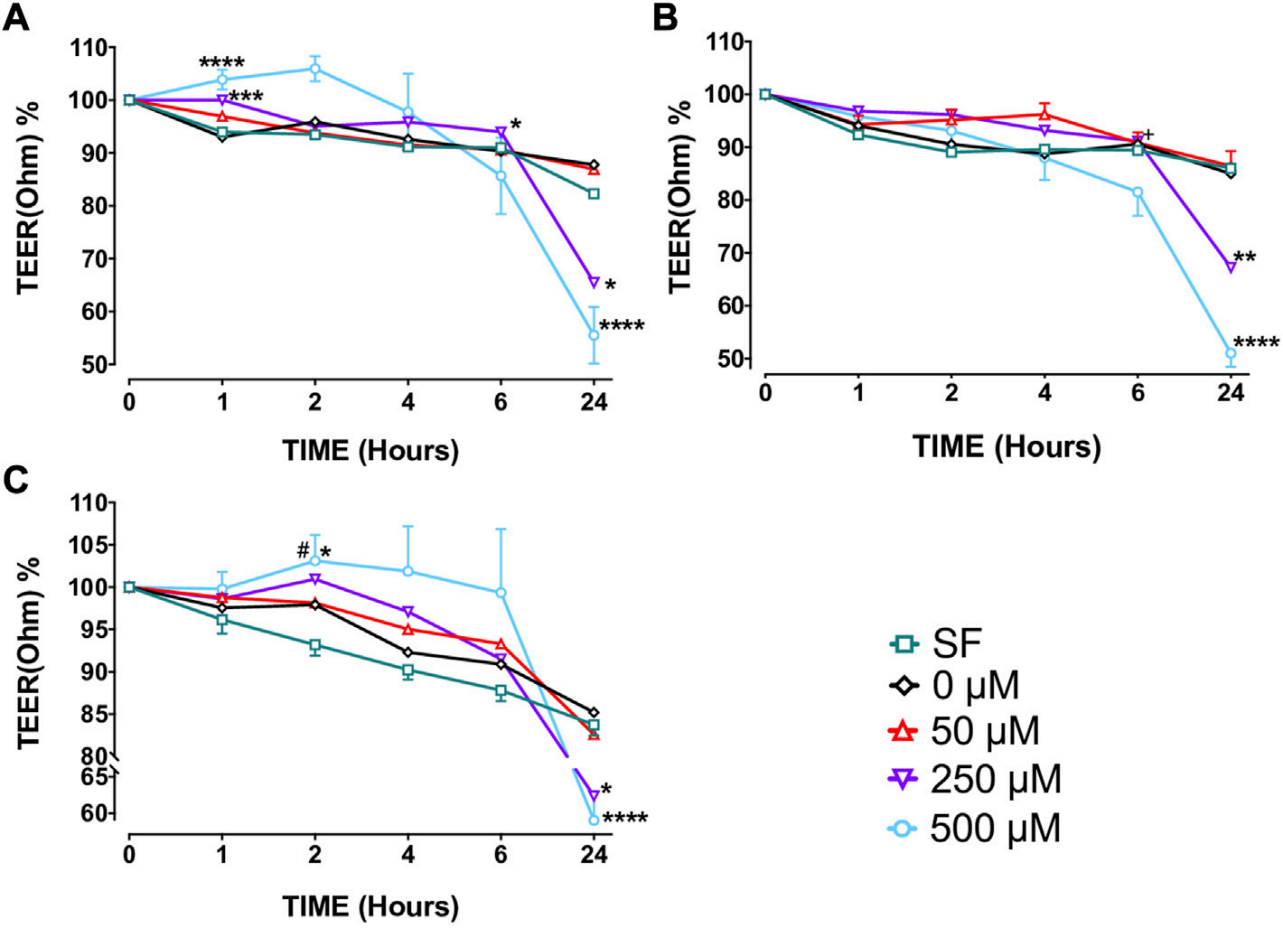
The effect of capsaicin on transepithelial electrical resistance (TEER) of bronchial epithelial cell (BEC) cultures of non-smokers **(A)**, smokers **(B)** and patients with COPD **(C)** Mean ± SEM is displayed for non-smoker (*N* = 5), smoker (*N* = 13) and patients with COPD (*N* = 7). (*p < 0.05, **p < 0.01, ***p < 0.001; ****p < 0.0001 vs. 0 μM capsaicin; +*p* < 0.05 vs. non-smokers, #*p* < 0.05 vs. smokers). SF, serum free treated cells; 0 µM indicates ethanol, solvent for capsaicin.

**FIGURE 3:**
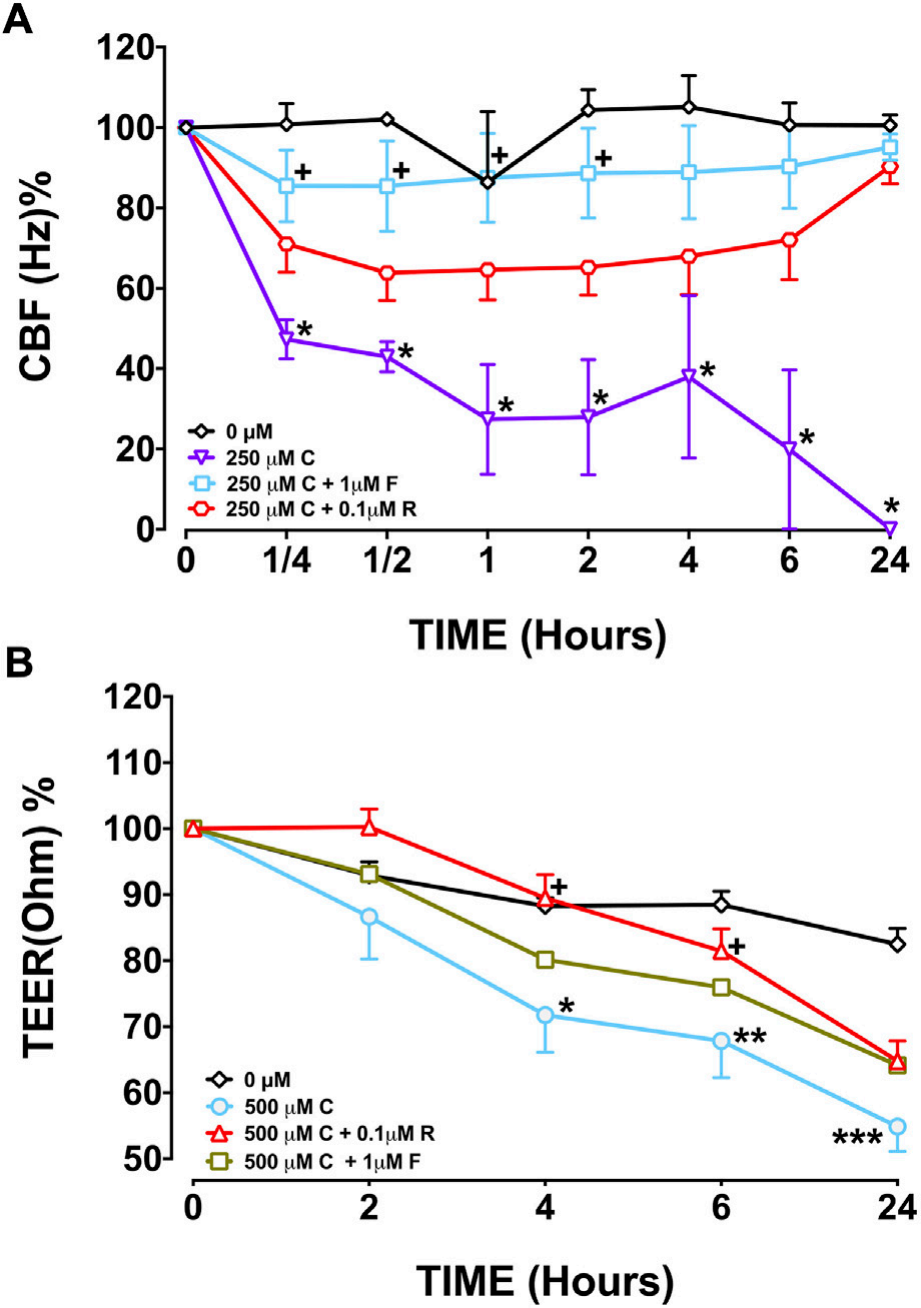
Effect of formoterol (F) and roflumilast (R) on CBF **(A)** and TEER **(B)** in bronchial epithelial cell (BEC) cultures of smokers incubated with 250 μM capsaicin (C) Mean ± SEM is displayed for smokers (*N* = 13). (Figure 3A: *p < 0.0001 vs. 0 μM capsaicin; +*p* < 0.05 vs. 250 μM capsaicin; Figure 3B: *p < 0.05, **p < 0.01, ***p < 0.001 vs. 0 μM capsaicin; +*p* < 0.05 vs. 500 mM capsaicin). 0 µM indicates DMSO, solvent for roflumilast and formoterol.

Capsaicin (250–500 μM) increased TEER of BECs from non-smokers at T1 (250 µM, *p* < 0.001; 500 µM, *p* < 0.0001) and T6 (250 µM, *p* < 0.05) but decreased TEER at T24 (250 µM, *p* < 0.05; 500 µM, *p* < 0.0001) (Figure 2A, Supplementary Table S2). Similarly, both 250 µM (*p* < 0.01) and 500 µM (*p* < 0.0001) capsaicin significantly decreased TEER in BEC cultures from smokers at T24 (Figure 2B, Supplementary Table S2). In COPD patients, a similar trend was observed, and the TEER was significantly suppressed by both 250 µM (*p* < 0.05) and 500 µM (*p* < 0.0001) capsaicin at T24 (Figure 2C, Supplementary Table S2). When culture groups were compared, 250 µM capsaicin-led decrease in TEER of smokers was significantly greater than that of non-smokers at T6 (*p* < 0.05), whereas COPD cultures had a higher TEER at T2 than cultures from smokers following exposure to 500 µM capsaicin (*p* < 0.05) (Figure 2, Supplementary Table S2). Roflumilast (0.1 μM) prevented effect of capsaicin on TEER at T4 (*p* < 0.05) and T6 h (*p* < 0.05), whereas formoterol (1 μM) showed no effect (Figure 3B).

### Effects of capsaicin on the release of GM-CSF and IL-8 from BECs

**FIGURE 4:**
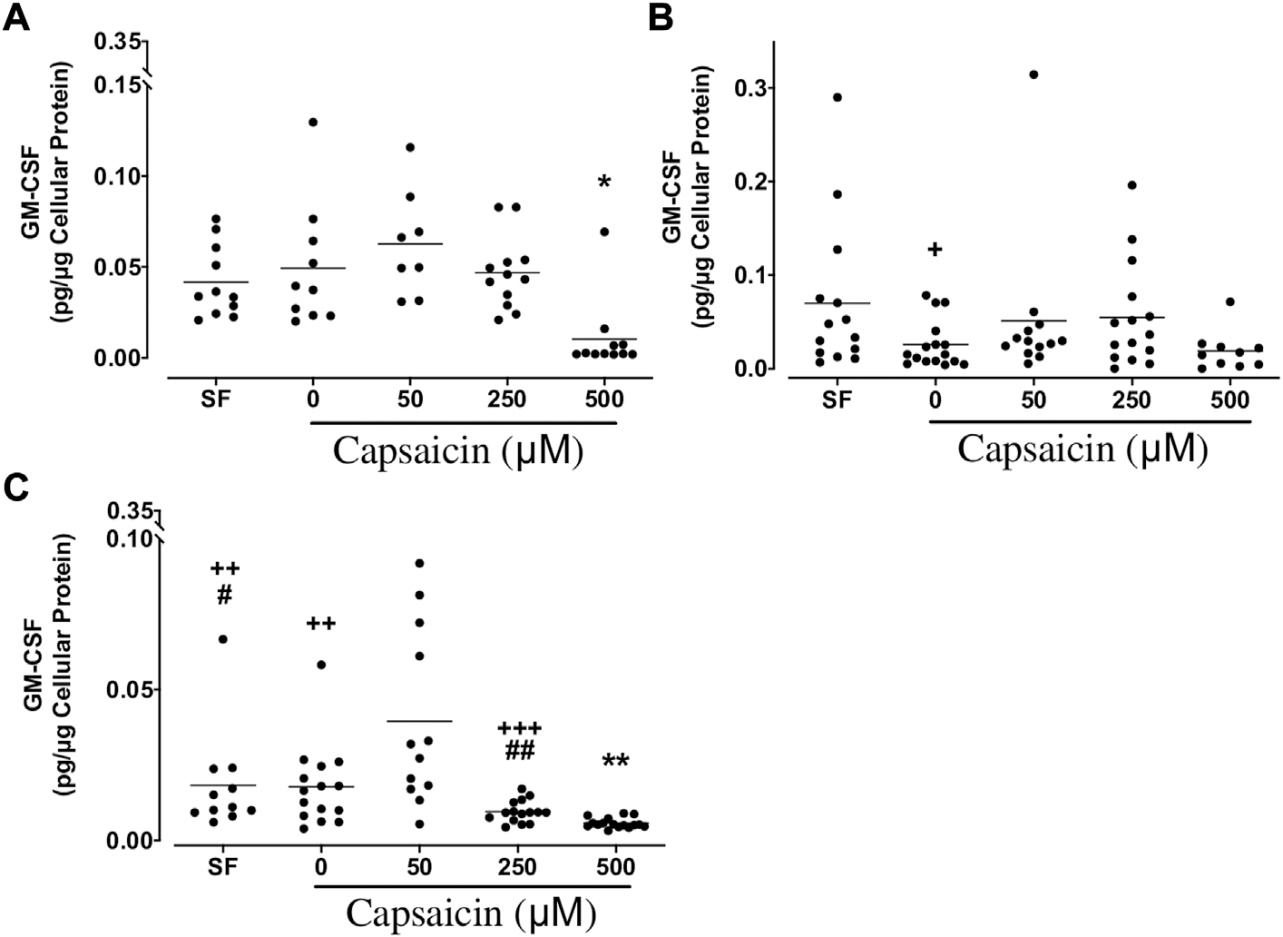
Effect of capsaicin on the release of granulocyte macrophage-colony stimulating factor (GM-CSF) of bronchial epithelial cells from non-smokers **(A)**, smokers **(B)** and patients with COPD **(C)**. Individual data points with means were displayed for non-smokers, smokers and patients with COPD (*p < 0.01, **p < 0.0001 vs. 0 μM capsaicin; +*p* < 0.05, ++*p* < 0.01, +++*p* < 0.001 vs. non-smokers; #*p* < 0.05, ##*p* < 0.01 vs. smokers). SF, serum free treated cells; 0 µM indicates ethanol, solvent for capsaicin.

*GM-CSF:* The highest concentration of capsaicin (500 µM) significantly decreased GM-CSF release from BEC cultures from both non-smokers (mean ± SEM = 0.010 ± 0.006 vs. 0.049 ± 0.011 pg/μg cellular protein; *p* < 0.05) and COPD patients (mean ± SEM = 0.0057 ± 0.0004 vs. 0.018 ± 0.003 pg/μg cellular protein; *p* < 0.01), while no significant change was found in cultures of smokers (Figure 4). As culture groups were compared, GM-CSF levels in 0 μM capsaicin-treated control cultures from smokers were significantly lower than those in non-smoker cultures (mean ± SEM = 0.026 ± 0.006 vs. 0.049 ± 0.011 pg/μg cellular protein; *p* < 0.05). Similarly, GM-CSF release in COPD BECs treated with SF, 0 and 250 μM capsaicin was significantly lower than that in BECs from non-smokers. Furthermore, GM-CSF release in COPD cells incubated in SF and 250 μM capsaicin was significantly decreased compared with that from cells from smokers (Figure 4).

**FIGURE 5:**
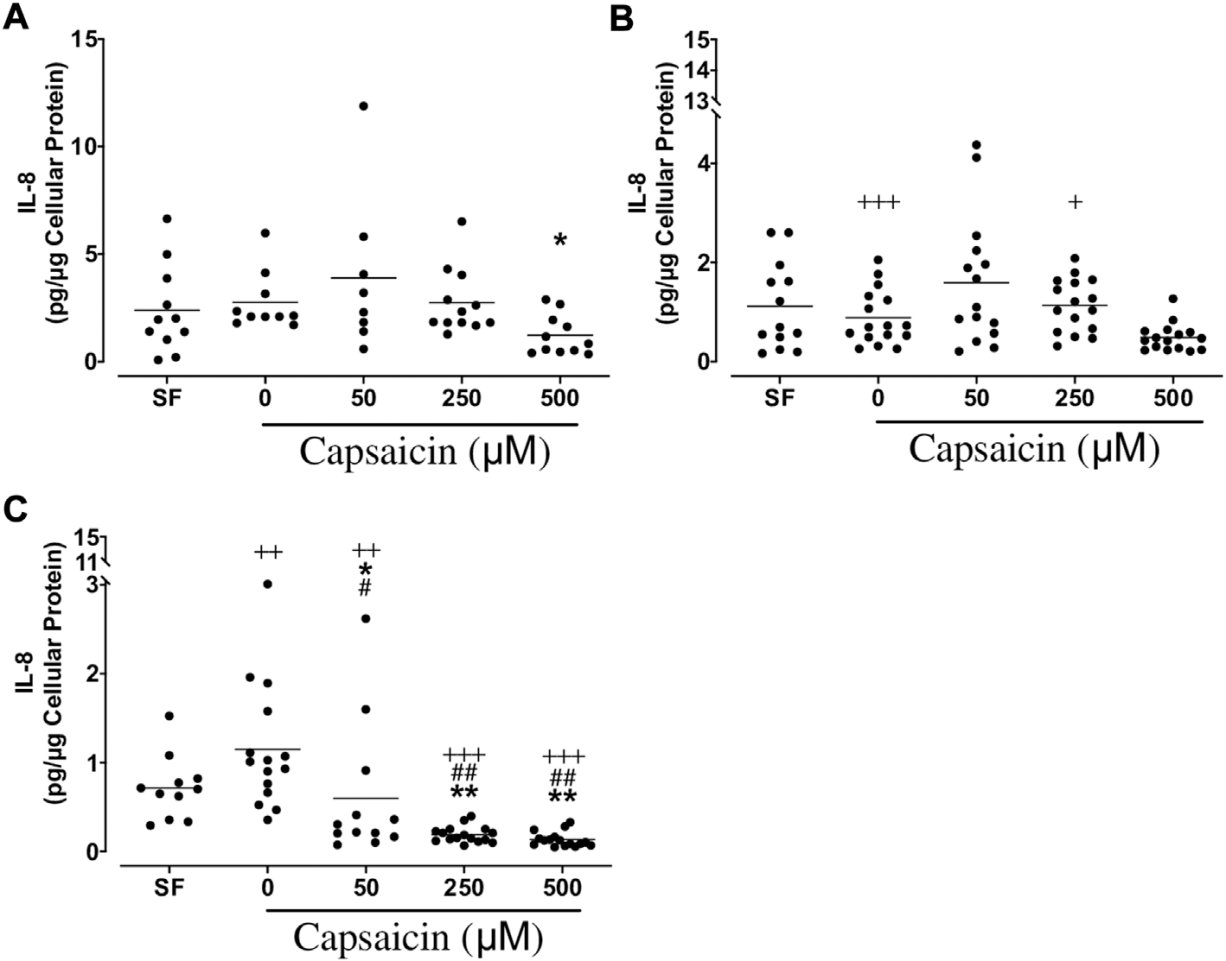
Effect of capsaicin on interleukin (IL)-8 release from bronchial epithelial cells of non-smokers **(A)**, smokers **(B)** and patients with COPD **(C)**. Individual data points with means were displayed for non-smokers, smokers and patients with COPD (*p < 0.05, **p < 0.0001 vs. 0 μM capsaicin; +*p* < 0.05, ++*p* < 0.01, +++*p* < 0.0001 vs. non-smokers; #*p* < 0.05, ##*p* < 0.001 vs. smokers). SF, serum free treated cells; 0 µM indicates ethanol, solvent for capsaicin.

*IL-8:* Capsaicin (500 µM) significantly suppressed IL-8 release from BECs from non-smokers (mean ± SEM = 1.22 ± 0.28 vs. 2.75 ± 0.43 pg/μg cellular protein; *p* < 0.05), whereas all doses of capsaicin [50 μM (mean ± SEM = 0.60 ± 0.22 vs. 1.15 ± 0.18 pg/μg cellular protein; *p* < 0.05), 250 μM (mean ± SEM = 0.19 ± 0.02 vs. 1.15 ± 0.18 pg/μg cellular protein; *p* < 0.01) and 500 μM (mean ± SEM = 0.13 ± 0.02 vs. 1.15 ± 0.18 pg/μg cellular protein; *p* < 0.01)] led to a significant decrease in the release of this cytokine from BECs of COPDs. However, capsaicin did not cause a significant change in IL-8 in smokers’ BECs. As study groups were compared, IL-8 from smoker BECs treated with 0 and 250 μM capsaicin was significantly decreased compared with that from non-smokers. Similarly, in COPD cells, capsaicin-induced IL-8 release was significantly lower than that from non-smokers’ BECs. Furthermore, IL-8 release by COPD cells treated with capsaicin was significantly lower than that from BECs of smokers (Figure 5).

### Effects of capsaicin on LDH release from BECs

**FIGURE 6:**
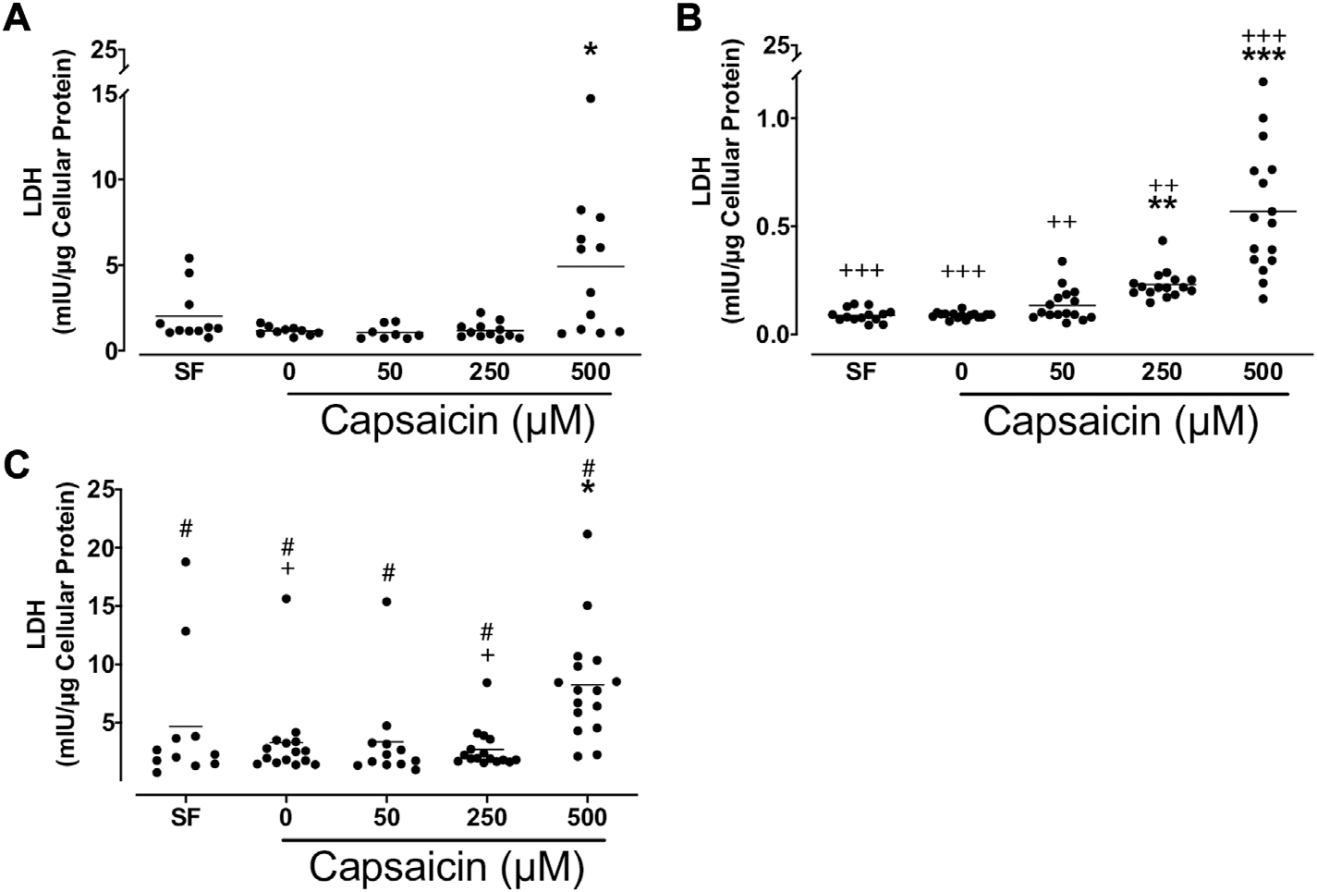
Effect of capsaicin **(C)** on the release of lactate dehydrogenase (LDH) of bronchial epithelial cells from non-smokers **(A)**, smokers **(B)** and patients with COPD **(C)**. Individual data points with means were displayed for non-smokers, smokers and patients with COPD (*p < 0.01, **p < 0.001, ***p < 0.0001 vs. 0 μM capsaicin; +*p* < 0.05, ++*p* < 0.01, +++*p* < 0.001 vs. non-smokers; #*p* < 0.0001 vs. smokers). SF, serum free treated cells; 0 µM indicates ethanol, solvent for capsaicin.

Capsaicin (500 μM) significantly increased LDH release from BECs from all groups of non-smokers (mean ± SEM = 4.9 ± 1.2 vs. 1.2 ± 0.1 mIU/μg cellular protein; *p* < 0.05), smokers (mean ± SEM = 0.57 ± 0.07 vs. 0.09 ± 0.00 mIU/μg cellular protein; *p* < 0.001) and patients with COPD (mean ± SEM = 8.2 ± 1.2 vs. 3.3 ± 0.9 mIU/μg cellular protein; *p* < 0.05), whereas 250 μM capsaicin only induced LDH release from BEC cultures from smokers (mean ± SEM = 0.23 ± 0.02 vs. 0.09 ± 0.00 mIU/μg cellular protein; *p* < 0.01; Figure 6). A comparison between cell groups demonstrated that BEC cultures from smokers had lower LDH release than non-smokers in all treated cultures. COPD cultures treated with both 0 and 250 μM capsaicin had a higher LDH release than those from non-smokers. Additionally, cultures from all COPD groups had higher LDH release than those from smokers (Figure 6).

## Discussion

In the present study, we investigated the effect of capsaicin on ciliary function, epithelial permeability, inflammatory cytokine release and cellular toxicity from BECs from well-characterized individuals of non-smokers, smokers, and COPD patients for the first time. High doses of capsaicin decreased CBF and TEER in a similar fashion in all study groups. Similarly, capsaicin decreased the release of GM-CSF and IL-8 while inducing LDH release in BECs, and this effect was more prominent in COPD cells. Formoterol and roflumilast partially prevented the decreasing effects of capsaicin on CBF and TEER. Our findings suggest that capsaicin can suppress ciliary activity and induce epithelial permeability and inflammatory changes while causing cellular toxicity in the bronchial epithelium and that formoterol and roflumilast may reduce some of these effects.

In the present study, we have demonstrated for the first time that capsaicin suppresses the CBF in human BEC cultures. A decrease in the CBF can impair mucociliary transport, possibly leading to the accumulation of toxic irritants in the lungs and a progressive decline in lung function. Ciliary dysfunction due to cellular and genetic factors can cause serious life-threatening health problems (Fahy and Dickey, 2010). Our CBF data confirmed by a previous study, OC decreased CBF of mouse tracheal rings that was inhibited by methylene blue and exogenous ATP (Delamanche et al., 2001). The authors suggested this inhibition to be due to the guanylate cyclase-dependent pathway, or protein kinase C-linked phosphorylation. Together, these studies suggest that capsaicin can inhibit the ciliary function of epithelial cells in both humans and animal models. Contrary to these findings, Eljamal and colleagues reported the activation of ciliary activity by capsaicin in the canine trachea (Eljamal et al., 1994). Hameister et al. found that capsaicin increased CBF in anaesthetized monkeys (Hameister et al., 1992). We believe the contrary views stem from the differences in dose and cell type vulnerability.

Interestingly, we found that formoterol, a selective ß2 receptor agonist that activates adenylyl cyclase, which in turn raises the level of intracellular cAMP in the cell (McCormack and Lyseng-Williamson, 2007), prevented the inhibitory effect of capsaicin on CBF. This suggests that capsaicin may decrease CBF by suppressing the activity of adenylyl cyclase intracellularly. In agreement with these findings, Benedetto et al. reported that a solution of cAMP significantly increased CBF of human nasal epithelial cells (Di Benedetto et al., 1991). Studies have also reported that other β2 agonists, such as salbutamol and salmeterol, increase CBF of human BECs (Devalia et al., 1992). Our findings suggest that formoterol may prevent the adverse effect of capsaicin on CBF in individuals exposed to tear gas preparations.

Although roflumilast, a selective PDE4 inhibitor (Lipari et al., 2013), tended to reverse the inhibitory effect of capsaicin on CBF of BECs, this was not significant. However, in *ex vivo* settings, where both PDE3 and PDE4 enzyme expression levels were prevalent, selective PDE4 inhibition caused efficient reversion in the CBF downregulation generated by cigarette exposure in both primary human BECs (Milara et al., 2012) and precision cut lung slices of mice (Milara et al., 2012; Zuo et al., 2018). Furthermore, formoterol enhanced the ability of roflumilast to prevent the decrease in CBF of human BECs exposed to cigarette smoke (Schmid et al., 2015). These findings indicate the need for further research concerning the interaction between these two drugs in the ciliary function of BECs. Unfortunately, we could not evaluate the effect of a combination of formoterol and roflumilast because of the limited number of cultures.

Preserving the structural features of the epithelial layer is critical to fulfil barrier function, thus maintaining the integrity of the airways. Increase in epithelial permeability provides the basis for the passage of inhaled irritants into the subepithelium and results in their harmful effects (Bayram et al., 2002). In the present study, we have demonstrated that capsaicin decreased the TEER of human BEC cultures obtained from subjects with and without COPD suggesting that this effect of capsaicin is not specific to the underlying pathology. However, our findings are the first to demonstrate these effects of capsaicin on human BECs. Previously, Tsukura et al. (Tsukura et al., 2007) showed that the TEER of gut epithelium (Caco-2 cells) was significantly decreased following exposure to capsaicin (100–300 µM).

Our findings demonstrated that roflumilast could prevent the capsaicin-induced decreases in TEER. Similarly, roflumilast prevented lung inflammation associated with increased expression of inflammatory cytokines (Konrad et al., 2015; Peng et al., 2019). In a rodent model, Wollborn et al. found that PDE4 inhibition by rolipram reduced microvascular permeability, caused by extracorporeal circulation-induced capillary leakage, by targeting endothelial cAMP (Wollborn et al., 2019). In a recent review by Mokra et al., various PDE3, PDE4, and PDE5 inhibitors were reported to stabilize the pulmonary epithelial-endothelial barrier and reduce sepsis- and inflammation-increased microvascular permeability (Mokra and Mokry, 2021).

In addition to ciliary function and epithelial integrity, we investigated whether capsaicin causes inflammatory changes in airway epithelial cells. Higher concentrations of capsaicin decreased the release of GM-CSF and IL-8, which play crucial roles in airway inflammation. Capsaicin-induced release of these cytokines from BECs of COPDs were lower than that from both smokers and non-smokers, suggesting that COPD cells are more sensitive to capsaicin. Various studies have reported the anti-inflammatory properties of capsaicin in different pathologies. It has been reported that capsaicin has anti-inflammatory properties in neurons, and that it could reduce inflammation in the salivary glands in humans and animal models by inhibiting the NF-κB pathway (Shin et al., 2016). In a rat model of pulmonary arterial hypertension (PAH), which was established by a single injection of monocrotaline, capsaicin pre-treatment reversed PAH, decreased the levels of macrophage/monocytes, and expression of inflammatory cytokines such as IL-1β, IL-6, and TNF-α in lungs. Furthermore, capsaicin decreased expression of phosphorylated-p38 (p-p38) MAPK, which was enhanced by monocrotaline (Xu et al., 2017). Similarly, capsaicin reduced levels of IL-6, TNF-α, and nitric oxide (NO) through suppression of the MAPK and NF-κB signalling pathways in mouse macrophages treated with LPS (Li et al., 2021). Although our findings of cytokine release appear to support these studies, it is also possible that the decreased levels of GM-CSF and IL-8 in our studies were caused by the toxic effects of capsaicin on BECs. Because higher concentrations of capsaicin (250–500 µM), which decreased the release of these cytokines, led to an increase in the release of LDH.

However, these findings have been paralleled by conflicting reports of harmful effects of high concentrations of capsaicin, and although some studies suggest that capsaicin activates different pathways at different concentrations in both human and rodent tissues (Fernandes et al., 2016), these mechanisms are yet to be elucidated. Studies have reported increased levels of plasma total IgE, eosinophils, and Th2 cytokines, such as IL-4, IL-5, and IL-13 in rats exposed to capsaicin (Han et al., 2017; Lee and Kuo, 2017; Chen et al., 2020). Xiang et al. reported that 40 mg/kg capsaicin did not show an adverse effect on gastrointestinal tissues, whereas higher doses of 60 and 80 mg/kg caused inflammation in jejunum, ileum and colon (Xiang, et al., Foods, 2022). *In vitro* studies found that capsanoids in pepper spray at 100 μM concentrations caused cellular death and increased IL-6 release from human lung epithelial cell lines (BEAS-2B and A549) and liver cell lines (HepG2) and that lung cells were more sensitive than liver cells (Reilly et al., 2003). The accountable toxic effect of capsaicin is the possible main reason for higher leukocyte count despite low IL-8 response shown by our results.

Mechanistic studies reported that capsaicin targeted ion channels transient receptor potential vanilloid type 1 (TRPV1) and transient receptor potential ankyrin 1 (TRPA1) localized in pain-sensing peripheral sensory neurons. These channels are shown to be linked to cough, asthma, lung injury, pain, dermatitis, itch, and neurodegeneration (Mukhopadhyay et al., 2016; Rothenberg et al., 2016). Furthermore, TRPA1 is thought to innervate respiratory tract starting from the oral cavity up to respiratory bronchioles, alveolar ducts and alveoli, and its activation leads to pulmonary inflammation, injury, remodelling and sensory symptoms like chronic cough in asthma, COPD, allergic rhinitis, and cystic fibrosis (Mukhopadhyay et al., 2016; Rothenberg et al., 2016). Additionally, capsaicin caused exogenous activation of lung nociceptors and increased CD45^+^ cells, including eosinophils, macrophages, and lymphocytes, in mouse lungs (Talbot et al., 2015).

Interestingly, it has been reported that the information regarding the interplay of TRPV1 activation and inflammation is contradictory (Munjuluri et al., 2021). This is thought to be related to the fact that TRPV1 activation can result in the release of either pro- or anti-inflammatory signaling molecules from nerve endings (Munjuluri et al., 2021). For example, capsazepine, an inhibitor of TRPV1 led to a decrease in the levels of LPS-induced IL6 release from cardiomyocytes, suggesting a role for TRPV1 activation in promoting LPS-induced cytokine release. In contrast, Wang et al. reported that capsaicin increased NO production and reduced inflammation in human umbilical vein endothelial cells (HUVECs) *via* TRPV1 activation. Capsaicin treatment also led to the attenuation of LPS-induced expression of endothelial adhesion molecules, activation of NF-κB, and monocyte adhesion in HUVECs that were also dependent on TRPV1 activation (Wang et al., 2017). Similarly, TRPV1 deficiency in mice was associated with an increase in proinflammatory markers such as TNF-α, IL-1β, and IL-6 suggesting anti-inflammatory properties of capsaicin (Munjuluri et al., 2021). However, none of these studies associated the contradictory effects (pro- or anti-inflammatory) of capsaicin with the dose utilized.

LDH is a marker of cellular toxicity because its release increases in damaged cells. High doses of capsaicin increased the release of LDH in BEC cultures obtained from all the study groups. These findings are in agreement with Isoda et al., who reported increased LDH release in gut Caco-2 cells following exposure to capsaicin (Isoda et al., 2001). Interestingly, capsaicin induced LDH release in COPD cells was higher than that from BECs from both non-smokers and smokers. This finding suggests that capsaicin directly causes toxicity in airway epithelial cells and that BECs from COPD patients may be more susceptible to the toxicologic effects of capsaicin.

The limitation of our study was that a relatively small number of donors (five patients) were available to produce the cultures in the nonsmoking group. In addition to this, formoterol and roflumilast studies were performed only in smokers because of challenges in obtaining adequate cultures from other study groups of non-smokers and COPDs. Therefore, whether the response of non-smokers and smokers with COPD would be different in their response to these drugs is unknown.

In conclusion, our study showed that high doses of capsaicin can impair the ciliary activity and integrity of the airway epithelium while changing the inflammatory response and inducing cellular toxicity, and that patients with COPD may be more susceptible to capsaicin. These effects are partially prevented by formoterol and roflumilast. Taken together, our findings demonstrate that components of tear gas such as capsaicin can cause adverse effects in the airways.

## Data Availability

The original contributions presented in the study are included in the article/Supplementary Material, further inquiries can be directed to the corresponding author.
