# Supplementary material for: Capsaicin suppresses ciliary function, while inducing permeability in bronchial epithelial cell cultures of COPD patients
Source: Front Pharmacol. 2022 Oct 6;13:996046. doi: 10.3389/fphar.2022.996046 (PMC9582664; doi:10.3389/fphar.2022.996046)
Supplement: Supplementary file 1 [file DataSheet1.docx]

**Capsaicin Suppresses Ciliary Function and Induces Permeability in COPD Bronchial Epithelial Cells**

Halil Ibrahim TOY^1,2,3,#^, Abdullah Burak YILDIZ^4,#^, Demet TASDEMIR KAHRAMAN^1,5^, Sedat ILHAN^1,6^, Oner DIKENSOY^7^, Hasan BAYRAM^1,7,8,9^*.

^1^Gaziantep University School of Medicine, Respiratory Research Laboratory, Gaziantep, Turkey

^2^Izmir Biomedicine and Genome Center, Izmir, Turkey

^3^Izmir International Biomedicine and Genome Institute, Dokuz Eylül University, Izmir, Turkey

^4^Koc University School of Medicine, Istanbul, Turkey

^5^ Gaziantep University, Faculty of Medicine, Department of Medical Biochemistry, Gaziantep, Turkey

^6^ Gaziantep University, Institute of Health Sciences, Department of Respiratory Biology, Gaziantep, Turkey

^7^Department of Chest Diseases, Gaziantep University School of Medicine, Gaziantep, Turkey

^8^Department of Pulmonary Medicine, Koc University School of Medicine, Istanbul, Turkey

^9^Koc University Research Centre for Translational Medicine (KUTTAM), Koç University, Istanbul, Turkey

# These authors contributed equally

* Corresponding author

**Correspondence:**

Koc University Hospital, Davutpasa cad. No:4, Topkapi, Zeytinburnu 34010, Istanbul, Turkey.

E-mail: habayram@ku.edu.tr

Tel: +90 850 250 8 250

Fax: F: +90 212 311 34 10

**Supplementary Material:**

**Materials and Methods**

***Study Patients***

Twenty-five volunteers (21 male and 4 female) with a mean age of 64 years (range: 11-80 years) participated in the study. Of these, 5, 13 and 7 volunteers were nonsmokers, smokers, and patients with COPD, respectively, **according to the guidelines from the Global Initiative for Obstructive Lung Disease (GOLD)** (1). The demographics of the study subjects are presented in Table 1. None of the volunteers had upper or lower respiratory tract infections within 1 month of the study. The study was approved by The Ethics Committee of Gaziantep University (2014/346), informed written consent was obtained from the study volunteers, and experiments adhered to the principles set out in the Declaration of Helsinki.

***Bronchial tissue***

Bronchial tissue was obtained from volunteers who had a lobectomy or pneumonectomy for various reasons, most commonly a tumour, at the Thoracic Surgery Clinic of Gaziantep University, Şahinbey Research and Training Hospital. The lung explant was transferred to the pathology laboratory immediately, and only tissue that appeared free of tumour and was considered normal by the pathologist was placed into ice-cold Medium 199 (Sigma, Inter lab, Turkey) and brought to the laboratory for tissue culture processing.

***Isolation, culture, and identification of primary bronchial epithelial cells***

The primary BEC cultures were obtained using an explant cell culture technique developed by Devalia et al. and described in detail elsewhere (2, 3). The epithelial layer was dissected from the underlying tissue using a dissection microscope, and then the epithelium was cut into 1-2 mm^3^ pieces. All the explants were washed with prewarmed and pregassed medium 199 containing 1% (vol/vol) antibiotics/antimycotic solution 3-4 times. For CBF studies, 4-5 explants were placed on 6-cm diameter Falcon® Primaria^TM^ plastic culture dishes (Becton Dickinson Biosciences, Turkey) or into 9-mm-diameter Falcon® Cell Culture Inserts with 0.45-mm pore size microporous membranes (Becton Dickinson) as air liquid interface (ALI) cultures for TEER studies. The explants were incubated in 5% CO_2_ in an air atmosphere at 37 °C in freshly prepared and micropore filter-sterilized medium 199 containing foetal calf serum (FCS), bovine pancreatic insulin, human transferrin, hydrocortisone, and antibiotic/antimycotic solution. Explants cultured in dishes were incubated in 2.0 ml of culture medium; for cultures grown in cellular culture inserts, 0.5 ml and 0.4 ml of medium were added to the insert and insert well, respectively, and the cells were grown to confluence within 2–3 weeks. The identity and purity of cells were checked in randomly selected cultures and confirmed by indirect immunoperoxidase staining techniques and light microscopy, as described previously (4).

***Preparation of capsaicin, formoterol and roflumilast solution***

Capsaicin was dissolved in ethanol (Sigma) and prepared in serum-free medium containing medium-199 and antibiotics/antimycotic (SF) at concentrations of 50, 125, 250 and 500 μM for experiments. Additionally, a control of 0.028% ethanol, which was the highest concentration used for 500 µM capsaicin in SF, was used as the negative control. Formoterol and roflumilast (Tocris, Medsantek, Turkey) were dissolved in dimethyl sulfoxide (DMSO). A solution containing the highest concentration of DMSO (0.015%) was used as the negative control. Formoterol (1 µM) and roflumilast (0.1 µM) were prepared at 250 µM and 500 µM in a solution containing capsaicin for CBF and TEER experiments, respectively.

***Measurement of the ciliary beat frequency in BECs incubated with capsaicin in the absence and presence of formoterol or roflumilast***

Two- to three-week cultures established on culture dishes were used for these studies, and CBF was measured using a Sisson-Ammons image analysis system (SAVA) (Ammons Engineering, Clio, MI, USA). Briefly, images obtained with magnification (×10) under the phase-contrast microscope were transferred to the computer monitor using a digital video camera. The CBF speed in the marked areas was recorded by selecting 30 frames per second at room temperature, 64 number fields, 0.469 frequency clarity, and 2.1 seconds of video length. Thus, the Hz value was obtained for each measurement. The culture was equilibrated for 2 min at room temperature and monitored for CBF ¼1/4, 0.5, 1, 2, 4, 6, and 24 h in the absence or presence of 50, 125 and 250 µM capsaicin, 1 µM formoterol or 0.1 µM roflumilast. The CBF in each culture was calculated as the mean of 5 areas within the culture at baseline and at each time point during incubation; the effect of treatment was expressed as the percentage change from the baseline.

***Measurement of transepithelial electrical resistance (TEER) in BECs incubated with capsaicin in the absence and presence of formoterol or roflumilast***

Confluent BEC cultures established under ALI conditions were used. First, the bronchial explants were removed, and the BECs in the inserts were allowed to grow over the area of the culture insert membrane that was left barren following the removal of the explant and when completely confluent cultures were used. The BEC cultures were then incubated for 24 hrs. in SF to equilibrate the cultures. The cultures were then gently washed 3 times with fresh, prewarmed and pregassed SF medium, and sets of at least 6 different cultures (each from a different subject) obtained from smokers and COPD patients were used for experiments. In the case of nonsmokers, sets of at least 5 different cultures were used. The cultures were equilibrated for 2 min at room temperature before measurement. The BEC cultures were incubated with 0, 50, 250, or 500 µM capsaicin in the absence and presence of 1 µm formoterol or 0.1 µM roflumilast, and TEER was measured at t0, t1, t2, t4, t6 and t24 h using an EVOM2™ microvolt-ohm metre (WPI world Precision Instruments, Germany). TEER was expressed as the percent change from baseline. At the end of the measurements, the media inside inserts and wells were collected and stored at -80 °C for the analysis of GM-CSF, IL-8 and LDH. The culture membranes were detached from the insert and stored at -80 °C for total protein analysis.

***Analysis of IL-8, GM-CS, and lactate dehydrogenase (LDH)***

The IL-8, GM-CSF and LDH levels were analysed using ELISA kits (R&D System Duoset, Starmed, Turkey) and LDH kits (Sigma) according to the manufacturer’s instructions. Samples were measured using a microplate reader (Bio - Tek) at 540-570 nm and were calculated using KC-Junior software (Bio - Tek). The levels of cytokines and LDH were expressed as pg of cytokine and milliunits (mIU) of LDH/µg of cellular protein.

***Total protein analysis***

Total proteins of the cells in the inserts were measured using a Qubit fluorometer (Invitrogen, Medsantek, Turkey) and the Qubit protein analysis kit (Invitrogen) according to the manufacturer’s instructions and were expressed as μg/ml.

***Statistical Analysis***

The data were tested for normality, and continuous variables were compared using one-way variance analysis, ANOVA/Dunnett’s multiple comparison tests or Kruskal–Wallis/Dunn’s multiple comparison tests. Unpaired t test or the Mann–Whitney U test was used to compare the individual treatment groups when needed. The results are expressed as means ± SEM or medians ± interquartile ranges (Q1 and Q3) and lower and upper ranges. P values smaller than 0.05 were regarded as significant. Statistical analysis was performed using PRISM version 8 (GraphPad Software Inc., San Diego, CA, USA).

**Supplementary Tables:**

**Table S1:** Effects of capsaicin on ciliary beat frequency (CBF) of bronchial epithelial cell (BEC) cultures from non-smokers, smokers, and patients with COPD.

**Non-smokers Smokers COPD**

| Time Periods(h) | SF/Capsaicin (µM) | Mean $\pm SEM$ | Mean $\pm SEM$ or Median ($IQR)$ | Mean $\pm SEM$ |
| --- | --- | --- | --- | --- |
| T1/4 | SF | NA | NA | NA |
|  | 0 | 114.7$\pm7.1$ | 103.4$\pm1.2$ | 102.3$\pm2$ |
|  | 50 | NA | NA | NA |
|  | 125 | NA | NA | NA |
|  | 250 | 78.0$\pm$8.5 | 73.1$\pm5.1$ | 82.0$\pm$4.3 |
| T1/2 | SF | NA | NA | NA |
|  | 0 | 113.3$\pm6.0$ | 104.1$\pm1.2$ | 104.5$\pm$2.8 |
|  | 50 | NA | NA | NA |
|  | 125 | NA | NA | NA |
|  | 250 | 71.6$\pm8.7$ | 61.06$\pm$4.1 | 67.0$\pm5.9$ |
| T1 | SF | NA | NA | NA |
|  | 0 | 118.8$\pm5.2$ | 99.0$\pm$2.0**^++^** | 104.9$\pm$2.9**#** |
|  | 50 | NA | NA | NA |
|  | 125 | NA | NA | NA |
|  | 250 | $69.2\pm7.9$ | 56.3 $\pm3.8$ | 53.6$\pm7.2$ |
| T2 | SF | 142.5$\pm$19.5 | $100.0\pm$1.7**^++++^** | 104.0$\pm$5.3 **###** |
|  | 0 | 119.6$\pm$6.3 | 101.9$\pm$1.9**^++^** | 107.1$\pm3.2$ |
|  | 50 | NA | 97.3$\pm$2.1 | 103.0$\pm$3.2 |
|  | 125 | 112.9$\pm6.9$ | 105.1$\pm1.5$ | 105.9$\pm5.5$ |
|  | 250 | $65.7\pm6.7$ ****** | 76.7$\pm7$.0******** | 58.6$\pm7.8$ ******* |
| T4 | SF | 156.9$\pm$0.1 | 96.2$\pm3.2$**^++++^** | $104.6\pm$6.1 **###** |
|  | 0 | 118.8±5.0 | 103.7(97.3-105.6) **^+^** | 105.4$\pm$3.6 |
|  | 50 | NA | 98.6 (91.9-103.1) | 100.7$\pm$4.0 |
|  | 125 | 114.6$\pm$4.5 | 106.7$\pm1.5$ | 111.6$\pm4.7$ |
|  | 250 | 60.5$\pm$6.9 ****** | 82.2$\pm$6.2 ***** | 58.8$\pm7.7$ ****** |
| T6 | SF | 160.7$\pm1.2$ | 99.6$\pm2.1$**++++** | 98.7$\pm$3.1 **####** |
|  | 0 | $121.6\pm6.5$ | 101.3(94.98-105.6)**^+^** | 105.4$\pm$3.2 **#** |
|  | 50 | NA | 98.8$\pm$2.4 | 102.7$\pm$4.1 |
|  | 125 | 113.3$\pm5.4$ | 104.7$\pm1.9$ | 108.0$\pm6.3$ |
|  | 250 | 40.5$\pm13.4$ ******* | 82.1$\pm$4.8 ****^,++^** | 58.7$\pm8.6$ ****** |
| T24 | SF | 140.0$\pm11.7$ | $95.4\pm2.6$**++++** | 88.1$\pm$2.5 **####** |
|  | 0 | 106.0$\pm11.3$ | 91.1$\pm$3.5 | 99.6$\pm$4.9 |
|  | 50 | NA | 85.9$\pm$3.4 | 96.6$\pm5.1$ |
|  | 125 | 82.8$\pm15.8$ | 94.3$\pm3.6$ | 68.3$\pm15.9$ |
|  | 250 | 0.1$\pm$0.01 ****** | 15.5$\pm$8.0 ******** | 0.1$\pm$0.0 ******** |

CBF, ciliary beat frequency; BEC, bronchial epithelial cell; COPD, chronic obstructive pulmonary disease; SEM, standard error of mean; IQR, interquartile range; SF, serum free treated cells; C, capsaicin treated cells; NS, not significant; NA, not available. *p<0.05, **p<0.01, ***p<0.001, and ****p<0.0001 versus 0μM capsaicin; +p<0.05, ++p<0.01, +++p<0.001, and ++++p<0.0001 versus non-smokers; #p<0.05, ###p<0.001, ####p<0.0001 versus smokers.

**Table S2:** Effects of capsaicin on transepithelial electrical resistance (TEER) of bronchial epithelial cell (BEC) cultures from non-smokers, smokers, and patients with COPD

**Non-smokers Smokers COPD**

| Time Periods(h) | SF/Capsaicin (µM) | Mean $\pm SEM$ or  Median ($IQR)$ | Mean $\pm SEM$ or  Median ($IQR)$ | Mean $\pm SEM$ or  Median ($IQR)$ |
| --- | --- | --- | --- | --- |
| T1 | SF | 93.9$\pm$1.8 | $92.4\pm$1.6 | 94.9(91.4-98.7) |
|  | 0 | 92.9$\pm$1.1 | 92.4(86.7-99.5) | 96.4$\pm$0.9 |
|  | 50 | 95.1$\pm$0.6 | 94.3$\pm$1.7 | 98.2(96.5-100.7) |
|  | 250 | 100.2$\pm$1.0 ******* | 96.9$\pm$1.3 | 99.7$\pm$1.3 |
|  | 500 | 101.7$\pm$1.8 ******** | 95.9$\pm$2.1 | 99.1$\pm$1.6 |
| T2 | SF | 93.5$\pm$1.7 | 89.1$\pm$2.0 | 93.7$\pm$1.3 |
|  | 0 | 95.5$\pm$1.0 | 90.6$\pm$2.1 | 95.8$\pm$1.0 |
|  | 50 | 93.8$\pm$1.2 | 93.2(90.8-98.8) | 97.3(87.9-96.5) |
|  | 250 | 98.3$\pm$1.4 | 96.2$\pm$1.4 | 100.2(93.6-107.5) |
|  | 500 | 98.6$\pm$2.8 | 93.1$\pm$2.7 | 102.5$\pm$2.4 *** #** |
| T4 | SF | 90.3$\pm$1.5 | 89.6$\pm$2.6 | 90.1$\pm$1.2 |
|  | 0 | 92.8$($88.7-96.0) | 88.7$\pm$1.8 | 92.1$\pm$1.0 |
|  | 50 | 91.1(90.0-93.5) | 96.2$\pm$2.1 | 92.3$\pm$1.1 |
|  | 250 | 99.8$\pm$1.8 | 95.7(89.3-99.9) | 97.1(91.4-104.2) |
|  | 500 | 92.3$\pm$5.2 | 88.0$\pm$4.2 | 99.6$\pm$4.3 |
| T6 | SF | 90.4$\pm$1.9 | 89.4$\pm$2.8 | 87.9$\pm$1.4 |
|  | 0 | 90.4$\pm$1.0 | 89.8(81.5-99.5) | 90.6$\pm$0.9 |
|  | 50 | 89.6$\pm$1.5 | 90.8$\pm2.0$ | 92.0$\pm$0.9 |
|  | 250 | 98.0$\pm$1.8 ***** | 91.1$\pm$1.7 **+** | 96.5$\pm$1.7 |
|  | 500 | 84.6$\pm$5.1 | 81.5$\pm$4.5 | 93.1$\pm$5.9 |
| T24 | SF | 81.0(79.1-85.1) | 80.2(73.7-93.5) | 83.9$\pm$1.4 |
|  | 0 | 86.5(84.2-89.0) | 85.0$\pm$2.4 | 85.1$\pm$1.4 |
|  | 50 | 85.9$\pm$1.6 | 86.4$\pm$2.8 | 83.9$\pm$2.1 |
|  | 250 | 74.4$\pm$4.0 ***** | 67.3$\pm$3.2 ****** | 73.0$\pm$3.5 ***** |
|  | 500 | 56.5$\pm$3.8 ******** | 49.5(37.8-63.4) ******** | 54.5$\pm$4.2 ******** |

TEER, Transepithelial electrical resistance; BEC, bronchial epithelial cell; COPD, chronic obstructive pulmonary disease; SEM, standard error of mean; IQR, interquartile range; SF, serum free treated cells; C, capsaicin treated cells; NS, not significant; NA, not available. *p<0.05, **p<0.01, ***p<0.001, ****p<0.0001 versus 0μM capsaicin; +p <0.05 compared to non-smokers; &p<0.05 compared to smokers.

**Table S3:** Effects of roflumilast and formoterol on CBF and TEER of BEC cultures from smokers

**CBF TEER**

| Time Periods(h) | Capsaicin ±  R (0.1) or F (1) (µM) | Mean $\pm SEM$ | Mean $\pm SEM$ |
| --- | --- | --- | --- |
| T1/4 | 0 | 100.8$\pm$5.2 |  |
|  | 250 | 47.3$\pm4.9$**** |  |
|  | 500 | NA |  |
|  | C+0.1 R | 71.1$\pm$7.1 |  |
|  | C+1 F | 85.5$\pm$9.0^+^ |  |
| T1/2 | 0 | 102.1$\pm$0.1 |  |
|  | 250 | 42.9$\pm3.8$**** |  |
|  | 500 | NA |  |
|  | C+0.1 R | 63.8$\pm6.8$ |  |
|  | C+1 F | 85.5$\pm11.3$^+^ |  |
| T1 | 0 | 86.4$\pm$17.6 |  |
|  | 250 | 27.4$\pm$13.6**** |  |
|  | 500 | NA |  |
|  | C+0.1 R | 64.6$\pm7.5$ |  |
|  | C+1 F | 87.6$\pm$11.0^+^ |  |
| T2 | 0 | 104.4$\pm5.1$ | 92.9$\pm2.1$ |
|  | 250 | 28.0$\pm$14.3**** | NA |
|  | 500 | NA | 86.6$\pm6.4$ |
|  | C+0.1 R | 65.2$\pm6.9$ | 100.2$\pm2.7$ |
|  | C+1 F | 88.6$\pm$11.2^+^ | 93.1$\pm4.3$ |
| T4 | 0 | 105.1$\pm7.9$ | 88.3$\pm1.3$ |
|  | 250 | 38.0$\pm20.2$**** | NA |
|  | 500 | NA | 71.8$\pm5.7$* |
|  | C+0.1 R | 68.0$\pm9.5$ | 89.5$\pm3.5$^#^ |
|  | C+1 F | 88.9$\pm$11.6 | 80.2$\pm3.6$ |
| T6 | 0 | 100.6$\pm$5.5 | 88.5$\pm$2.1 |
|  | 250 | 19.9$\pm$19.8**** | NA |
|  | 500 | NA | 67.8$\pm5.6$** |
|  | C+0.1 R | 72.1$\pm9.9$ | 81.5$\pm3.4$^#^ |
|  | C+1 F | 90.3$\pm$10.4 | 75.9$\pm2.7$ |
| T24 | 0 | 100.6$\pm$2.6 | 82.5$\pm2.4$ |
|  | 250 | 0.12$\pm$0.01**** | NA |
|  | 500 | NA | 54.9$\pm3.8$*** |
|  | C+0.1 R | 90.3$\pm4.3$ | 64.8$\pm3.0$ |
|  | C+1 F | 95.1$\pm$3.2 | 64.1$\pm3.2$ |

CBF, ciliary beat frequency; TEER, transepithelial electrical resistance; BEC, bronchial epithelial cell; SEM, standard error of mean; IQR, interquartile range; SF, serum free treated cells; C, capsaicin treated cells; NA, not available. *p<0.05, **p<0.01, ***p<0.001, and ****p<0.0001 versus 0μM capsaicin; +p<0.05 versus 250 μM capsaicin; #p<0.05 versus 500 µM capsaicin.

**Figure S1.**

**Figure S2.**

**Supplementary Figure Legends:**

Figure S1: Effects of 250 µM capsaicin on ciliary beat frequency (CBF) of bronchial epithelial cell cultures of non-smokers (A), smokers (B) and COPD patients (C) at T¼, T½, T1, T2, T4, T6, and T24 h. Mean± SEM is displayed for non-smoker (N=5), smoker (N=13) and COPD (N=7). (*p<0.01, **p<0.001, ***p<0.0001 vs 0 μM capsaicin; +p<0.05, ++p<0.01 vs non-smokers, #p<0.05 vs smokers).

Figure S2: Comparison of CBF of BEC cultures of non-smokers, smokers and COPD groups in SF. Mean± SEM is displayed for non-smoker (N=5), smoker (N=13) and COPD (N=7). (*p<0.01 vs non-smokers). SF, serum free treated cells.

**References:**

1. GOLD**.** GLOBAL STRATEGY FOR PREVENTION, DIAGNOSIS AND MANAGEMENT OF COPD: 2022 Report. Global Initiative for Chronic Obstructive Lung Disease; 2022:1-177.

2. Devalia JL, Sapsford RJ, Wells CW, Richman P, Davies RJ**.** Culture and comparison of human bronchial and nasal epithelial cells in vitro. Respir Med. 1990;84(4):303-12.

3. Gogebakan B, Bayraktar R, Ulaslı M, Oztuzcu S, Tasdemir D, Bayram H**.** The role of bronchial epithelial cell apoptosis in the pathogenesis of COPD. Mol Biol Rep. 2014;41(8):5321-7.

4. Bayram H, Devalia JL, Sapsford RJ, Ohtoshi T, Miyabara Y, Sagai M, et al. The effect of diesel exhaust particles on cell function and release of inflammatory mediators from human bronchial epithelial cells in vitro. Am J Respir Cell Mol Biol. 1998;18(3):441-8.
